# Wind stilling shapes grassland water use efficiency by enhancing soil moisture retention

**DOI:** 10.1126/sciadv.aee4995

**Published:** 2026-05-13

**Authors:** Haohao Wu, Congsheng Fu, Philippe Ciais, Zelalem A. Mekonnen, Lingling Zhang, Qing Zhu, Jiafu Mao, Jianyao Chen, Dagang Wang, Guishan Yang

**Affiliations:** ^1^ Key Laboratory of Lake and Watershed Science for Water Security, Nanjing Institute of Geography and Limnology, Chinese Academy of Sciences, Nanjing 210008, China.; ^2^ Collaborative Innovation Center on Forecast and Evaluation of Meteorological Disasters (CIC-FEMD), Nanjing University of Information Science & Technology, Nanjing, China.; ^3^ Le Laboratoire des Sciences du Climat et de l’Environnement, IPSL-LSCECEA/CNRS/UVSQ Saclay, Gif-sur-Yvette, France.; ^4^ Climate and Ecosystem Sciences Division, Lawrence Berkeley National Laboratory, Berkeley, CA 94720, USA.; ^5^ School of Geographic Information and Tourism, Chuzhou University, Chuzhou 239000, China.; ^6^ Environmental Sciences Division, Oak Ridge National Laboratory, Oak Ridge, TN 37830, USA.; ^7^ School of Geography and Planning, Sun Yat-sen University, Guangzhou 510275, China.; ^8^ College of Geography and Remote Sensing, Hohai University, Nanjing 210098, China.

## Abstract

Covering more than 40% of Earth’s vegetated surface, grasslands critically regulate terrestrial carbon and water cycles. Their ecosystem water use efficiency (WUE_eco_), the ratio of carbon uptake to water loss, governs drought resilience in these water-limited ecosystems. While global terrestrial wind speed declined substantially from the 1960s to 2000s followed by a recovery in the subsequent decade, the extent and mechanisms of its influence on grassland WUE_eco_ remain poorly understood. Using site observations, satellite data, Earth system models, and wind manipulation experiments, we found a consistent negative sensitivity of grassland WUE_eco_ to wind speed. Mechanistically, declining winds reduce evaporative water loss, improve soil moisture, and promote stomatal opening, thereby enhancing carbon uptake. Wind speed changes accounted for 7.7 to 25.7% of WUE_eco_ increases under historical and future climates, making wind the second most important climatic driver after atmospheric carbon dioxide. Since Earth system models underestimate observed wind speed declines, future WUE_eco_ increases and drought resilience may exceed current projections for global grassland.

## Teaser

Declining wind speed boosts carbon uptake while conserving water, enhancing drought resilience in global grasslands.

## INTRODUCTION

Grasslands, covering more than 40% of Earth’s vegetated surface, play a pivotal role in regulating terrestrial carbon-water feedbacks and are highly sensitive to climate change (*1*, *2*). Grasslands ecosystems are generally water limited, especially in arid and semiarid regions, and ecosystem water use efficiency (WUE_eco_; the ratio of carbon uptake to water loss) is a critical determinant of their resilience to drought (*3*, *4*). To date, rising atmospheric carbon dioxide (CO_2_) concentration is known to enhance WUE_eco_ by optimizing stomatal conductance (*5*, *6*), whereas increasing atmospheric drought, commonly measured by vapor pressure deficit (VPD), suppresses WUE_eco_ by intensifying water stress (*7*). However, the role of wind speed, as another key climatic factor, in global carbon-water coupling has been largely overlooked, although it plays a crucial role in regulating water availability and influencing plant physiological responses (*8*–*10*).

It is unexpected that wind speed as a climatic driver in shaping WUE_eco_ has received limited attention, especially given the marked changes in global wind speed over recent decades. Observations have revealed a 30-year decline in terrestrial surface wind speed starting from 1980s, known as “terrestrial stilling” (*9*, *11*), which was followed by a transient recovery after 2010 (*12*). However, Coupled Model Intercomparison Project Phase 6 (CMIP6) models project a renewed decline in wind speed under future climates (*13*, *14*). Grasslands, in particular, have experienced more pronounced reductions in wind speed during the stilling period compared to other ecosystems (fig. S1). Unlike tall forests, where increases in vegetation growth and surface roughness introduce complex feedbacks and complicate causal links with wind speed decline (*15*), the low stature of grassland vegetation minimizes such biophysical interactions. This allows wind speed changes to exert more direct and unidirectional effects on ecosystem water and carbon fluxes. Although a recent study convincingly showed that declining wind speed enhanced gross primary productivity (GPP) across global terrestrial ecosystems through alterations in stomatal conductance (*14*), existing studies on wind speed impacts have largely focused on one aspect, either the carbon or the water cycle. As a result, the impact of wind speed changes on WUE_eco_, a key metric of carbon-water coupling, across the water-limited global grasslands remains largely unquantified.

The mechanistic pathways through which wind affects WUE_eco_ are also poorly understood. Wind alters leaf boundary layer conductance and leaf-air vapor gradients, thereby modulating soil evaporation, canopy interception evaporation, and plant transpiration (*8*–*10*, *16*). Previous reviews have qualitatively highlighted wind’s influence on plant morphology and ecological function (*8*, *10*). Recent studies have further suggested that historical wind speed decline has contributed to carbon uptake (*14*), vegetation growth (*17*), and delayed leaf senescence (*18*). However, critical knowledge gaps remain in partitioning the effects of wind speed changes on WUE_eco_. Many statistical efforts to isolate effects of wind often lack methodological robustness, suffering from issues such as weak causal inference and limited mechanistic validation (*19*, *20*). Field experiments that manipulate wind as a single varying factor, similar in design to warming or free-air CO_2_ enrichment (i.e., FACE) experiments, represent the gold standard for isolating wind effects and uncovering the underlying mechanisms (*21*). However, technical constraints have made wind manipulation in field settings exceedingly difficult. These knowledge gaps are likely to widen under future climate conditions, in which declining wind speeds are projected to coincide with rising atmospheric CO_2_ concentrations, increasing VPD, shifting precipitation regimes, and intensifying soil dying, leading to complex interactions that remain poorly understood.

To address these critical knowledge gaps, we integrate observations from 991 meteorological stations and 13 eddy covariance flux towers with multiple reanalysis datasets and CMIP6 simulations spanning historical and future Shared Socioeconomic Pathways (SSP) emission scenarios. We apply robust statistical frameworks to isolate the effect of wind speed on WUE_eco_ and validate the inferred mechanisms with wind speed manipulation experiments using the Community Land Model version 5 (CLM5). We aim to (i) quantify the sensitivity of WUE_eco_ to wind speed changes across global grasslands, (ii) elucidate the mechanistic pathways through which wind speed regulates WUE_eco_, and (iii) evaluate the contribution of wind speed changes relative to other climatic drivers to WUE_eco_ increases under both historical and future climates. By integrating multiple lines of evidence from observational, reanalysis, modeling, and experimental perturbation, our study provides essential insights into the role of wind speed in regulating carbon-water coupling within grassland ecosystems and its importance in predicting ecosystem responses to a changing climate.

## RESULTS AND DISCUSSION

### Decadal variations in wind speed and WUE_eco_ anomalies

**Fig. 1.**
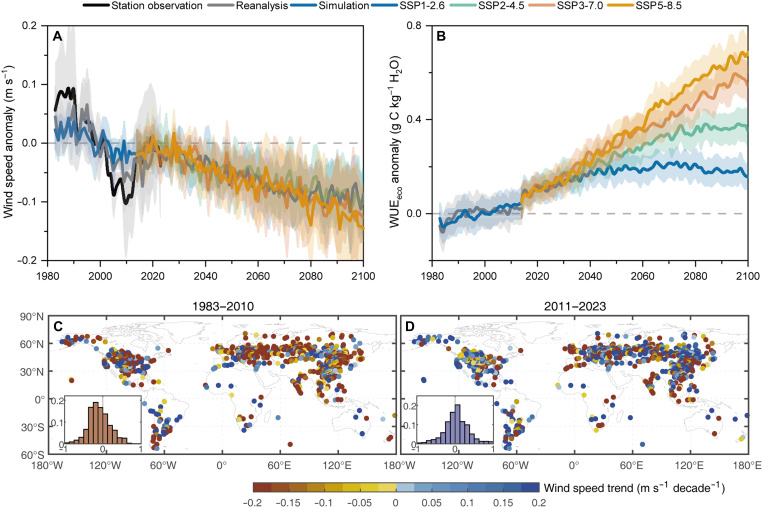
Anomalies in wind speed and ecosystem water use efficiency (WUE_eco_) over global grasslands. (**A**) Spatially averaged wind speed anomalies (thick lines) derived from 991 meteorological stations, reanalysis datasets (CRU, ERA5, and MERRA2), and historical (1983–2014) and future (2015–2100) CMIP6 model simulations under four emission scenarios. (**B**) Spatially averaged WUE_eco_ anomalies (thick lines), derived from reanalysis data–based WUE_eco_ and from historical and future CMIP6 model simulations under four emission scenarios. Shaded areas represent ±1 SD. (**C** and **D**) Spatial distributions of observed wind speed trends during 1983–2010 (C) and 2011–2023 (D) across 991 grassland meteorological stations. Insets show the corresponding probability density distributions of wind speed trends (*x* axis, wind speed trend; *y* axis, probability density).

From 1983 to 2100, wind speed anomaly across global grasslands follows a clear triphasic trajectory, characterized by a persistent decline during 1983–2010, a transient recovery during 2011–2028, and an intensified projected decline for 2029–2100 (Fig. 1A; see Materials and Methods for turning point detection and the exclusion of fire-affected and human-modified regions). Observations from 991 grassland stations in the Hadley Centre’s Integrated Surface Database (HadISD) reveal a substantial wind speed decline of −0.072 m s^−1^ per decade (averaging −2.15% per decade relative to the mean) during 1983–2010, with 61.49% of stations exhibiting statistically significant negative trends (Fig. 1C). A similar pattern emerges from three independent reanalysis datasets—Climatic Research Unit Japanese Reanalysis (CRU JRA) v2.2, fifth generation European Centre for Medium-Range Weather Forecasts Re-Analysis (ERA5), and Modern-Era Retrospective Analysis for Research and Applications Version 2 (MERRA2; average −0.051 m s^−1^ per decade; −1.61% per decade; 62.8% of pixels) and from six CMIP6 models capable of reproducing the triphasic trajectory (average −0.025 m s^−1^ per decade; −0.82% per decade; 78.61% of pixels; Fig. 1A and fig. S2). Following this period of decline, wind speed anomaly experienced a brief reversal—averaging 0.041, 0.032, and 0.011 m s^−1^ per decade across observations, reanalysis, and CMIP6 simulations, respectively—before resuming a projected long-term decline after 2028 (Fig. 1A). The magnitude of this future decline depends on emission pathways, with the fraction of pixels exhibiting negative trends increasing from 61.32% under SSP1-2.6 (−0.010 m s^−1^ per decade), 64.86% under SSP2-4.5 (−0.011 m s^−1^ per decade), 73.88% under SSP3-7.0 (−0.013 m s^−1^ per decade), to 74.98% under SSP5-8.5 (−0.015 m s^−1^ per decade; Fig. 1A and fig. S3).

The three-phase trajectory of wind speed in grasslands aligns with the broad temporal patterns identified across all terrestrial biomes (*14*). However, the fluctuations are markedly more pronounced in grassland ecosystems. For example, the rate of wind speed decline during 1983–2010 was substantially higher in grasslands than in other terrestrial biomes (fig. S1). This is consistent with earlier reports indicating that historical wind stilling was particularly pronounced in the mid-latitudes of the Northern Hemisphere (*9*, *11*), where most grasslands are located (Fig. 1, C and D). Moreover, the recovery in wind speed across grasslands since the 2010s and the subsequent renewed decline after 2029, as identified in this study, are consistent with earlier findings across all terrestrial ecosystems in the northern mid-latitudes and globally (*12*–*14*, *22*). Notably, the magnitude of both decreasing and increasing wind speed trends is systematically reduced from observations to reanalysis to CMIP6 simulations, in agreement with previous reports (*13*, *23*), suggesting that Earth system models may underestimate future wind-driven ecological changes. This convergence across datasets and alignment with previous studies lend confidence to our identification of a three-phase wind trajectory specific to global grasslands.

In contrast to wind speed, WUE_eco_ anomaly across grasslands shows an upward trend from 1983 to 2100 (Fig. 1B and fig. S4). During 1983–2014, the WUE_eco_ trend derived from reanalysis datasets (average 0.019 g C kg^−1^ H_2_O per decade; 85.65% of pixels showing a positive trend) was generally consistent with the trend derived from CMIP6 simulations (average 0.029 g C kg^−1^ H_2_O per decade; 89.55%). Future projections suggest continued increases in WUE_eco_ anomaly, with trends scaling positively with anthropogenic emission intensity. Specifically, projected trends range from 0.010 g C kg^−1^ H_2_O per decade under SSP1-2.6 (87.85% of pixels showing a positive trend), to 0.037, 0.061, and 0.077 g C kg^−1^ H_2_O per decade under SSP2-4.5 (95.73%), SSP3-7.0 (98.64%), and SSP5-8.5 (99.24%), respectively.

Long-term increases in WUE_eco_ have been widely attributed to rising atmospheric CO_2_ (*5*, *6*, *24*). However, early Moderate Resolution Imaging Spectroradiometer (MODIS)-based estimates reported declining WUE_eco_ trends across global grasslands during 2001–2013 (*25*). Such discrepancies likely arise from structural omissions in the MODIS light use efficiency (LUE) framework, which lacks an explicit mechanism for CO_2_ fertilization (*26*). Beyond these algorithmic uncertainties, WUE_eco_ anomalies exhibit pronounced temporal variability marked by a post-2000 plateau (Fig. 1B). This pattern likely reflects a growing constraint whereby rising VPD offsets CO_2_-induced gains (*7*), consistent with recent global flux network (FLUXNET)-based evidence from grassland sites predominantly monitored in the post-2000 era (*27*). Crucially, our results reveal that concurrent declines in wind speed—a frequently overlooked factor—further shaping the WUE_eco_ dynamics, motivating the sensitivity analyses and mechanistic modeling presented below.

### Sensitivity of WUE_eco_ to wind speed changes over past decades

**Fig. 2.**
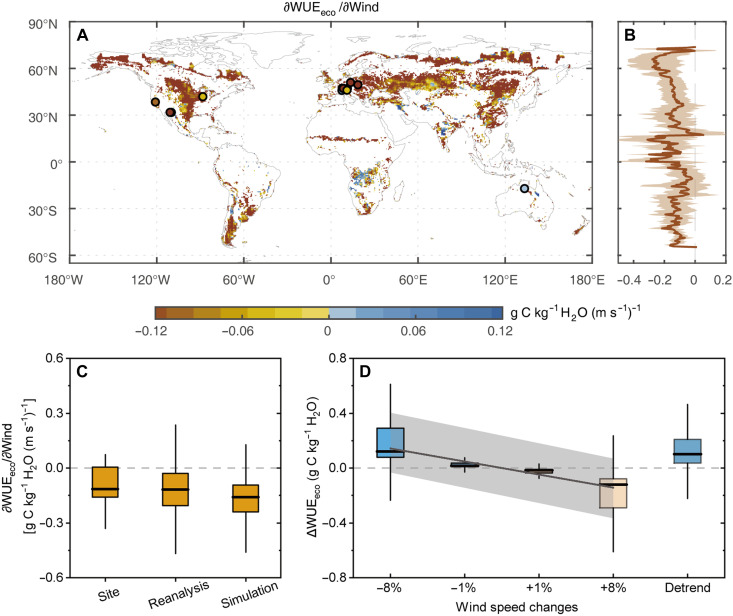
Sensitivity of WUE_eco_ to wind speed changes across grasslands during 1983–2014. (**A**) Spatial distribution of the sensitivities of WUE_eco_ to wind speed changes (∂WUE_eco_/∂Wind), averaged values derived using the PCR approach based on both reanalysis datasets and CMIP6 model simulations. Colorful scatters denote corresponding estimates from four FLUXNET sites (see table S1). (**B**) Latitudinal distribution of ∂WUE_eco_/∂Wind, where thick line represents the ensemble mean and shaded areas represent ±1 SD. (**C**) Statistical distributions (boxplots) of ∂WUE_eco_/∂Wind derived from FLUXNET site observations, reanalysis datasets, and CMIP6 simulations. (**D**) WUE_eco_ changes induced by wind speed perturbations in CLM5 experiments (−8, −1, +1, and +8% wind speed perturbations). “Detrend” represents the WUE_eco_ changes resulting from removing the historical wind speed trend, calculated as the difference between the reference and detrended experiments. In (C) and (D), the central horizontal lines represent the median, box edges indicate the interquartile range (IQR; 25th to 75th percentiles), and whiskers extend to 1.5 times the IQR. The solid line in (D) represents a linear regression fit, with the gray shaded area indicating the 95% confidence interval.

Using a principal components regression (PCR) approach to minimizing collinearity among climatic factors (see Materials and Methods), we identified a predominantly negative sensitivity of WUE_eco_ to wind speed (∂WUE_eco_/∂Wind) across global grasslands during 1983–2014 (Fig. 2, A to C). Specifically, 80.26 and 86.16% of grassland pixels exhibited negative ∂WUE_eco_/∂Wind values based on the annual-scale reanalysis datasets and CMIP6 simulations, respectively (fig. S5). The spatial median values of ∂WUE_eco_/∂Wind for 1983–2014 were −0.12 (± 0.19, spatial SD), −0.13 (± 0.17), and −0.16 (± 0.19) g C kg^−1^ H_2_O (m s^−1^)^−1^ based on eddy covariance site observations, reanalysis datasets, and CMIP6 simulations, respectively (Fig. 2C and table S1). These results were further corroborated by mechanistic evidence from wind manipulation experiments using the CLM5 model. From CLM5 simulations, a uniform 1% increase in wind speed across grassland pixels reduced the median WUE_eco_ by 0.015 g C kg^−1^ H_2_O, while an +8% increase resulted in a larger reduction of 0.118 g C kg^−1^ H_2_O. Conversely, wind speed reductions of −1 and −8% increased WUE_eco_ by 0.017 and 0.121 g C kg^−1^ H_2_O, respectively (Fig. 2D; the range of −8 to 8% is selected to encompass the observed rates of decadal wind speed changes; see Materials and Methods). The simulated WUE_eco_ responded approximately linearly and negatively to changes in wind speed (Fig. 2D). Furthermore, removing the wind speed decline trend during 1983–2010 in the detrended simulation resulted in a reduction of 0.101 g C kg^−1^ H_2_O in the median WUE_eco_ relative to the control run (Fig. 2D). Together, both statistical estimates and process-based simulations consistently revealed a pronounced negative sensitivity of WUE_eco_ to wind speed.

### Mechanisms underlying wind speed effects on WUE_eco_

**Fig. 3.**
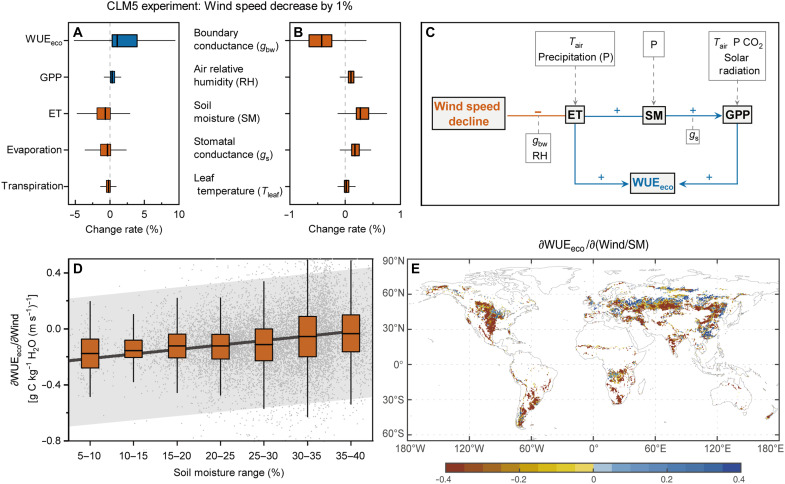
Mechanisms underlying the influences of wind speed change on WUE_eco_. (**A** and **B**) Percentage changes of intermediate variables induced by 1% decrease in wind speed, derived from CLM5 experimental simulations. (**C**) Pathways by which wind speed decline affects WUE_eco_, derived from SEM analyses based on CLM5 experimental simulations. (**D**) Changes in ∂WUE_eco_/∂Wind across different SM ranges. The solid line represents a linear regression fit, with the gray shaded area indicating the 95% confidence interval. (**E**) Spatial distribution of the sensitivity of WUE_eco_ to interactions between wind speed and soil moisture [∂WUE_eco_/∂(Wind/SM)]. Results in (D) and (E) are derived from reanalysis datasets. In (A), (B), and (D), the central horizontal lines within boxes represent the median, box edges indicate the IQR (25th to 75th percentiles), and whiskers extend to 1.5 times the IQR.

Building on the CLM5 wind manipulation experiments, we quantified the aerodynamic and physiological pathways through which wind speed modulates WUE_eco_. A 1% decline in wind speed enhanced GPP by 0.31 ± 0.44%. Meanwhile, evapotranspiration (ET) was reduced by 0.65 ± 0.89%, primarily due to suppressed soil evaporation (−0.37 ± 0.54%) and transpiration (−0.17 ± 0.21%) (Fig. 3A). These shifts were accompanied by decreased boundary layer conductance to water vapor (*g*_bw_; −0.43 ± 0.41%), enhanced stomatal conductance (+0.18 ± 0.10%), elevated air relative humidity (+0.10 ± 0.08%), increased soil moisture (SM; +0.38 ± 0.16%), and negligible changes in leaf temperature (+0.02 ± 0.05%) (Fig. 3B). To disentangle the feedback loops among wind speed, GPP, ET, and their associated drivers, we used structural equation modeling (SEM), a robust method for testing causal relationships, to examine the interactions and feedback mechanisms among these variables. SEM analyses revealed that wind speed reduction suppressed ET through two synergistic pathways. First, it directly decreased *g*_bw_, which limited atmospheric vapor exchange. This first-principles pathway is further supported by the aerodynamic scaling between wind speed (*U*) and *g*_bw_, as well as by the significant positive response of Δ*g*_bw_ to the first-order theoretical predictor 0.5 *U*^−1/2^Δ*U* (fig. S6). Second, it indirectly elevated air humidity thereby reducing evaporative demand (Fig. 3C). These two effects of wind speed change outweighed the impacts of enhanced stomatal conductance and suppressed ET. Suppressed ET helped retain SM, which, in turn, promoted stomatal opening and enhanced GPP.

Our findings indicate a dominant regulatory role of soil water feedbacks in mediating the WUE_eco_ response to wind speed changes. Across global grassland pixels, the sensitivity of WUE_eco_ to wind speed (∂WUE_eco_/∂Wind) showed a significant positive relationship with SM (*r* = 0.22; *P* < 0.001; *n* = 10,763); ∂WUE_eco_/∂Wind became increasingly negative as SM declined from 40 to 5% (Fig. 3D). This pattern was further supported by eddy covariance observations at two representative FLUXNET grassland sites (CH-Fru and US-Var), where ∂WUE_eco_/∂Wind during drought periods was more negative than estimates derived from the full observational record (fig. S7). Across individual drought events, ∂WUE_eco_/∂Wind showed a strong positive correlation with mean SM (*r* > 0.61). To further capture this interaction, we incorporated an interaction term between wind speed and soil moisture (Wind/SM) into the PCR framework (the rationale for using Wind/SM rather than their product is detailed in Materials and Methods). Results indicated that 75.77% of grassland pixels exhibited negative sensitivities to this interaction [∂WUE_eco_/∂(Wind/SM); Fig. 3E], highlighting a reinforcing feedback in which SM depletion amplified the regulatory effect of wind speed on WUE_eco_.

The present study demonstrates that wind speed decline enhances WUE_eco_ by simultaneously suppressing ET and promoting carbon uptake. This finding aligns with established physiological principles whereby lower wind speeds reduce evaporation (*28*) and transpiration (*16*) while enhancing terrestrial vegetation carbon uptake (*14*), plant growth (*17*), and delaying leaf senescence (*18*). Previous studies have typically examined only one aspect of wind speed impacts, either on the carbon cycle or on the water cycle. By contrast, using the robust attribution framework (e.g., PCR and CLM5 experiments) developed in our prior work (*14*), we investigated how wind speed changes influence carbon-water coupling, specifically WUE_eco_. WUE_eco_ has ecological implications that differ fundamentally from those of the carbon or water cycle because it reflects assimilated carbon per unit of water loss and governs ecosystem resilience to drought. This metric is particularly important for water-limited grasslands, where wind speed declines are most pronounced (fig. S1). Furthermore, our results uncover a previously unrecognized “drought-resistance buffer” mechanism: Increases in WUE_eco_ driven by wind speed reductions intensify under drier SM conditions. Although a short-term, leaf-level field experiment reported increased WUE under high wind speeds due to convective cooling under intense radiation (*29*), such conditions are rare and extreme, and the study did not account for ecosystem-scale effects of wind speed changes on WUE. Notably, while our site-level results provide essential empirical validation, the number of publicly available grassland eddy covariance sites that meet the stringent data requirements of this study remains limited. This constraint underscores a critical gap in current observational networks and highlights the urgent need for expanded long-term flux observations to better constrain the global sensitivities of WUE_eco_ to wind speed changes and to further elucidate the underlying mechanisms.

### Sensitivity of WUE_eco_ to wind speed under future climate

**Fig. 4.**
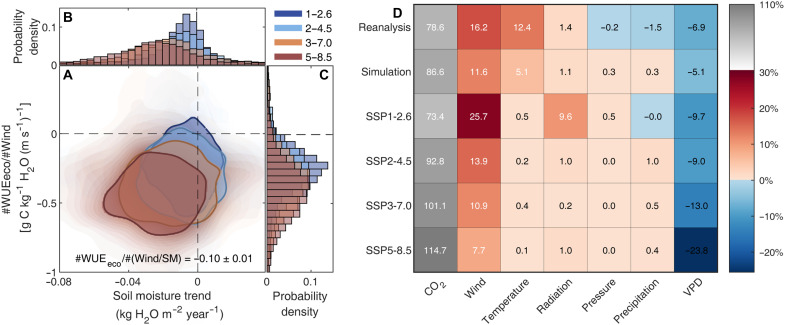
Changes in the sensitivity of ecosystem water use efficiency to wind speed (∂WUE_eco_/∂Wind) and relative contributions of climatic factors. (**A** to **C**) Joint distributions of ∂WUE_eco_/∂Wind and SM trends under four future emission scenarios, with corresponding probability density distributions shown for each axis. The coefficient ∂WUE_eco_/∂(Wind/SM) in (A) denotes the sensitivity of WUE_eco_ to the interactions between wind speed and SM across the four scenarios, expressed as the median value ±1 SD. (**D**) Relative contributions (%) of climatic factors (CO_2_, wind speed, air temperature, solar radiation, air pressure, precipitation, and VPD) to the increases in WUE_eco_, estimated from reanalysis datasets and CMIP6 simulations for the historical period (1983–2014) and future projections (2015–2100) under the four emission scenarios.

CMIP6 simulations show that grassland WUE_eco_ becomes increasingly sensitive to wind speed under all future emission pathways (∂WUE_eco_/∂Wind; Fig. 4A). The fraction of grassland pixels exhibiting negative ∂WUE_eco_/∂Wind increases from 86.65% under SSP1-2.6 to 89.20% under SSP5-8.5, while the median sensitivity value intensifies from −0.22 g C kg^−1^ H_2_O (m s^−1^)^−1^ under SSP1-2.6 to −0.27, −0.31, and −0.34 under SSP2-4.5, SSP3-7.0, and SSP5-8.5, respectively (fig. S8). These projected sensitivities are more negative than the historical range of −0.16 (derived from CMIP6 simulations) to −0.12 (derived from flux site observations; Fig. 2C), indicating a future strengthening of wind regulation on WUE_eco_. This strengthening in wind sensitivity is accompanied by widespread soil drying (Fig. 4A). The fraction of grassland pixels showing declining SM trends increases from 76.05% under SSP1-2.6 to 84.89% under SSP5-8.5, and the median drying rates rise from −0.009 to −0.035 kg H_2_O m^−2^ year^−1^ (Fig. 4A and fig. S9). Consistent with historical analyses, negative sensitivities of ∂WUE_eco_/∂(Wind/SM) of −0.10 ± 0.01 were identified, covering 66.11 to 74.96% of global grassland pixels under future scenarios (Fig. 4A and fig. S10), confirming that drier soils amplify the negative effect of wind speed on WUE_eco_.

Collectively, our results provide robust evidence that WUE_eco_ exhibits negative sensitivity to wind speed across global grasslands during both historical and future climates, with SM acting as a key modulator. Notably, historical soil drying has been widespread, and future projections under high-emission scenarios indicate further intensification (*30*). This soil drying amplifies the negative sensitivity of WUE_eco_ to wind speed, thereby reinforcing the increase in WUE_eco_ under declining wind speeds in grassland ecosystems. The synergistic effects of wind speed and soil water on WUE_eco_ indicate that grassland carbon-water coupling increasingly depends on interactions between atmospheric and soil processes. Such dependence highlights the risk that compounding effects may intensify under concurrent climate drivers such as elevated atmospheric CO_2_, higher air temperatures, and altered precipitation regimes (*3*, *5*, *7*, *30*). Thus, beyond establishing the sensitivity of WUE_eco_ to wind speed and elucidating its underlying mechanisms, we further quantify the relative contribution of wind speed changes to historical and projected increases in grassland WUE_eco_, providing a comparative perspective against other climatic drivers.

### Contribution of wind speed changes to the WUE_eco_ increases

We estimated the wind-induced change in WUE_eco_ by multiplying the PCR-derived sensitivity of WUE_eco_ to wind speed (i.e., ∂WUE_eco_/∂Wind) with the long-term trend in wind speed (see Materials and Methods). Across global grasslands, wind speed changes result in increases in WUE_eco_ during both historical (1983–2014) and CMIP6 projection periods (2015–2100) (fig. S11). The magnitude and spatial distribution of wind-induced WUE_eco_ increases during 1983–2014 were broadly consistent between reanalysis datasets and CMIP6 simulations. Their median values were 0.0108 and 0.0112 g C kg^−1^ H_2_O, respectively, with positive values covering 79.26 and 82.16% of global grassland pixels. This agreement, however, partly reflects a coincidence, as CMIP6 models underestimated historical wind speed anomalies (Fig. 1A) but overestimated the negative sensitivity of WUE_eco_ to wind speed (Fig. 2C). During 2015–2100, the median wind-induced WUE_eco_ reached 0.0139, 0.0111, 0.0223, and 0.0422 g C kg^−1^ H_2_O under SSP1-2.6, SSP2-4.5, SSP3-7.0, and SSP5-8.5 emission scenarios, respectively, with positive values covering 78.39 to 80.20% of grassland pixels.

On the basis of the same procedure, we found that wind speed changes emerged as the second-largest contributor to WUE_eco_ increases—surpassed only by rising atmospheric CO_2_, among the climatic drivers considered (atmospheric CO_2_ concentration, air temperature, shortwave radiation, air pressure, precipitation, and VPD) during both historical (1983–2014) and CMIP6 projection periods (2015–2100). The proportional increase in WUE_eco_ driven by wind speed changes (7.7 to 25.7%; Fig. 4D) is greater than the corresponding wind speed–induced increase in global GPP reported in our previous work (6.0 to 17.7%) (*14*). This difference is reasonable, considering that wind speed decline enhances WUE_eco_ not only through promoting GPP but also by suppressing ET, and the combined effects are therefore larger than its effect on carbon flux alone. Furthermore, grasslands experience more pronounced SM limitations than other ecosystems (e.g., forests), which further enhances the regulatory importance of wind speed in these water-limited biomes.

Although the absolute wind-induced WUE_eco_ increase was greater under high-emission scenarios (fig. S11), its relative contribution declined from SSP1-2.6 (25.7%) to SSP5-8.5 (7.7%) due to the amplified role of rising CO_2_ under high emission scenario. In contrast, VPD consistently contributed negatively (−5.1 to −23.8%), and this effect intensified under higher-emission scenarios (Fig. 4D). Our results are consistent with previous studies that show increases in WUE_eco_ from rising CO_2_ (*5*, *6*, *24*) while a declining WUE_eco_ from rising VPD (*7*). Together, these findings indicate that wind speed changes represent a critical and previously undervalued regulator of ecosystem carbon-water dynamics, particularly under moderate emission scenarios where CO_2_-driven effects are less overwhelming.

We note that quantifying the relative contribution of wind speed changes to WUE_eco_ increases depends on the uncertainties in wind speed and other climatic datasets among site observations, reanalysis products, and CMIP6 models (Fig. 1A). These differences reflect both observational constraints (*9*, *11*, *12*) and methodological uncertainties in measuring and accurately modeling wind speed (*31*). Future projections are also subject to uncertainties from CMIP6 models’ structure and parameterization. To reduce this uncertainty, we selected CMIP6 models capturing historical wind dynamics reasonably well. Notably, although CMIP6 models capture the divergent effects of CO_2_, wind speed, and VPD on WUE_eco_ dynamics, they overestimate the CO_2_ fertilization effect by ~8% (Fig. 4D). This bias suggests that WUE_eco_ gains under future high-emission scenarios may be more modest than recently simulated (Fig. 1B). Under low-emission pathways (i.e., SSP1-2.6), WUE_eco_ could reach a tipping point and decline earlier than projected. Nevertheless, the identified negative sensitivity of WUE_eco_ to wind speed is robust across multiple independent lines of evidence, including datasets from site observations, reanalysis products, and CMIP6 simulations, as well as complementary statistical and model experimental approaches. Last, although our model experiments capture the negative sensitivity of WUE_eco_ to wind speed and elucidate the underlying mechanisms, further field observations, particularly controlled wind experiments, are needed to broaden research on wind-induced effects on global grassland ecosystems.

## MATERIALS AND METHODS

### Estimation of WUE_eco_

We calculated ecosystem-level monthly water use efficiency (WUE_eco_; unit: g C kg^−1^ H_2_O) as the ratio of GPP to ET, which differs from the leaf-scale calculation methods (*32*). This metric is widely used to assess ecosystem carbon-water coupling and its response to climate changes (*7*, *24*, *33*). Unlike subdaily WUE_eco_ estimates, which require VPD corrections to account for the nonlinear GPP-ET relationship, our monthly-scale estimation did not require such adjustments (*34*, *35*). Although rainy days may affect WUE_eco_ estimates (*32*), excluding them was impractical at the monthly resolution used in this study.

### Data sources

In this study, we integrated site observations, reanalysis datasets, and CMIP6 simulations to investigate the spatiotemporal dynamics of wind speed and WUE_eco_ and to quantify the sensitivity of WUE_eco_ to wind speed changes (∂WUE_eco_/∂Wind).

#### Site observations

We obtained wind speed data from the HadISD (v.3.4.1.2024f) global database (*36*), distributed by the UK Met Office Hadley Centre. This dataset includes records from 9957 stations worldwide that have undergone rigorous quality control to eliminate errors while preserving extreme values (*36*). We selected only grassland stations that met the following criteria: continuous data coverage from 1983 to 2023, complete records for all 12 calendar months in each year, and a minimum of 15 valid observation days per month. After applying these filters, 991 stations remained for assessing wind speed dynamics; however, we excluded these sites from ∂WUE_eco_/∂Wind estimation due to the absence of corresponding WUE_eco_ observations.

We used GPP and ET data from the FLUXNET2015 tier 1 dataset to analyze ∂WUE_eco_/∂Wind at the site scale. In total, we analyzed 124 site-years of data (table S1). This dataset underwent standardized quality control and gap-filling procedures (*37*). From this dataset, GPP was estimated from measured net ecosystem exchange (NEE) using a nighttime-based flux partitioning algorithm (*38*). ET was derived from measured latent heat flux (LE) using a conversion coefficient between energy and ET (*39*). Additional environmental variables—including wind speed, surface air temperature, air pressure, precipitation, incoming shortwave radiation, VPD, and atmospheric CO_2_ concentration—were also extracted. To ensure the physical reliability of WUE_eco_ and avoid bias from mathematical artifacts (e.g., when ET approaches zero during dormant seasons), we applied a two-step filtering procedure: (i) excluding days with negligible water fluxes (ET < 0.05 mm day^−1^) and (ii) removing WUE_eco_ outliers that exceeded the 99th percentile of the distribution at each site. We selected 13 grassland sites (table S1) based on continuous NEE, LE, and climatic observations for at least 7 years, as well as statistical significance (*P* < 0.001) in subsequent PCR analyses.

#### Reanalysis datasets

We used a reanalysis-based WUE_eco_ dataset, which was produced via machine learning upscaling of FLUXNET eddy covariance observations (*7*). In this approach, global gridded estimates of GPP and ET were first generated for 1982–2016. ET was simulated using machine learning models guided by the Penman-Monteith equation and driven by satellite-derived leaf area index and climate reanalysis variables (e.g., air temperature, precipitation, air pressure, humidity, radiation, and wind speed). For GPP, the machine learning simulation was guided by a LUE modeling framework (*40*) and driven by satellite-derived fraction of absorbed photosynthetically active radiation, PAR, air temperature, VPD, and a static plant functional types (PFT)-specific maximum LUE (maxLUE) parameter. The maxLUE parameterization was derived from a flux tower–based optimization of maxLUE (*41*). WUE_eco_ was subsequently calculated as the ratio of GPP to ET and validated against site-level FLUXNET observations, demonstrating statistically significant predictive skill (*P* < 0.001). Across all global sites, *R*^2^ values were 0.74 for site-year comparisons and 0.57 for interannual trends. For grassland sites—the primary focus of this study—*R*^2^ values were 0.80 for site-year comparisons and 0.57 for interannual trends (*7*).

Global gridded environmental variables were compiled from multiple reanalysis sources: air temperature, air pressure, and precipitation from the Climatic Research Unit Time-Series (CRU TS) v4.0.4 (*42*); wind speed, shortwave radiation, and specific humidity from the CRU JRA v2.2 (*43*); two additional wind speed products from the ERA5 (*44*) and the MERRA2 (*45*); atmospheric CO_2_ concentrations from Cheng *et al.* (*46*); and SM (0 to 28 cm) from ERA5. VPD was calculated using the Buck (*47*) equation based on air temperature, air pressure, and relative humidity from the CRU TS dataset.

#### CMIP6 model simulations

We extracted historical (1983–2014) and future (2015–2100) GPP, ET, and relevant environmental variables from simulations of six CMIP6 models [Australian Community Climate and Earth System Simulator Earth System Model version 1.5 (ACCESS-ESM1-5), Canadian Earth System Model version 5 (CanESM5), Community Earth System Model Version 2 (CESM2), Centro Euro-Mediterraneo sui Cambiamenti Climatici Earth System Model version 2 (CMCC-ESM2), Geophysical Fluid Dynamics Laboratory Earth System Model version 4 (GFDL-ESM4), and Institute of Numerical Mathematics Climate Model version 5.0 (INM-CM5-0)] (*48*). Future projections were conducted under four emission scenarios, including the SSP1-2.6, SSP2-4.5, SSP3-7.0, and SSP5-8.5. We selected the CMIP6 models based on data availability and their ability to reproduce the observed wind speed dynamics over global grasslands, especially the post-2010 recovery pattern (Fig. 1A and fig. S12). SM for the 0- to 28-cm depth was calculated as a thickness-weighted mean by averaging the soil layers within this range according to each model’s individual layer thickness.

### Identification of global grasslands and high-disturbance regions

We delineated global grasslands using the Historical Land-Cover Change and Land-Use Conversions Global Dataset, available from National Oceanic and Atmospheric Administration National Climatic Data Center. To minimize anthropogenic disturbances, we excluded regions with a Human Footprint Index > 50%, annual burn area > 30%, or cultivated and managed vegetation coverage > 30% (*49*), using the Global Human Footprint Dataset (Last of the Wild Project, Version 2) and the Global Fire Emissions Database 4 for burned areas (*50*).

### Identification of turning points in wind speed trend

Previous studies have identified 2010 as the turning year in global terrestrial wind speed trends, marking a shift from long-term decline to an increasing trend (*12*, *51*). Our observations, reanalysis datasets, and CMIP6 simulations confirm this turning point for global grasslands (Fig. 1A and fig. S12) (*52*). Furthermore, CMIP6 projections suggested a second turning point after the 2010s, where wind speed is expected to revert to a declining trend (Fig. 1A). To identify this turning point, we first conducted a series of linear regression analyses for each future emission scenario (SSP1-2.6, SSP2-4.5, SSP3-7.0, and SSP5-8.5). We varied the starting year from 2010 to 2080 while keeping the end year fixed at 2100 and calculate the wind speed trend for each period. The starting year associated with the most negative trend was identified as the likely onset of the next decline phase. The identified turning points were 2026, 2025, 2033, and 2028 for the four scenarios (SSP1-2.6, SSP2-4.5, SSP3-7.0, and SSP5-8.5), respectively, with an average of 2028. To ensure statistical rigor, we further validated these dates using segmented regression (piecewise linear regression), which identifies breakpoints by minimizing the residual sum of squares. The segmented regression yielded turning points of 2031, 2025, 2039, and 2019 for the four scenarios, respectively, with a multiscenario mean of 2028.5. On the basis of the consistent results obtained from both methods, we partitioned the wind speed trend over global grasslands into three phases: 1983–2010 (historical decline), 2011–2028 (reversal), and 2029–2100 (projected future decline).

### Estimating the sensitivity of WUE_eco_ to wind speed and its contribution to WUE_eco_ increases

To quantify the sensitivity of WUE_eco_ to wind speed (∂WUE_eco_/∂Wind), we used a PCR approach to mitigate multicollinearity among climatic variables by transforming them into uncorrelated principal components (*14*, *53*, *54*). The climatic variables included wind speed, air temperature, air pressure, precipitation, shortwave radiation, VPD, and CO_2_ concentration. We estimated ∂WUE_eco_/∂Wind for individual FLUXNET sites, each grassland pixel from reanalysis datasets and CMIP6 simulations. For reanalysis estimates, we calculated three independent ∂WUE_eco_/∂Wind values using wind speed data from CRU, ERA5, and MERRA2, with the final estimate obtained by averaging. To ensure the reliability of this ensemble approach, we compared the ∂WUE_eco_/∂Wind derived from each individual dataset. The results exhibited high consistency across the three products, with global median (± SD) sensitivities of −0.13 (±0.19) for CRU, −0.14 (±0.21) for ERA5, and −0.10 (±0.11) g C kg^−1^ H_2_O (m s^−1^)^−1^ for MERRA2 (fig. S13). This cross-dataset agreement underscores that the captured wind-WUE_eco_ relationship is a robust physical signal rather than an artifact of a specific meteorological product. Similarly, for CMIP6 simulations, we derived estimates for each model under historical and future scenarios and then averaged across models.

The PCR framework follows the robust attribution protocol validated in our prior study (*14*). At each observation site or pixel, WUE_eco_ (*y*) and seven climatic variables (*x*_j_: *x*_1_ to *x*_7_) were first standardized via *z*-score normalization as

$$y^{*}=\frac{y-\text{ym}}{\text{ys}}$$ (1)

$$x_{j}^{*}=\frac{x_{j}-{\text{xm}}_{j}}{{\text{xs}}_{j}}$$ (2)

where y^*^ and *x*_j_^*^ are normalized *y* and *x*_j_, respectively; ym and xm_j_ are the mean values of *y* and *x*_j_, respectively; ys and xs_j_ are the SDs of *y* and *x*_j_, respectively.

The principal components analysis was then applied to the normalized climatic variables (*x*_j_^*^) to transform them into a set of orthogonal components. These results, including the spatial loadings and variance explained by each component, are presented in fig. S14. This step effectively orthogonalized the climatic drivers to account for their inherent physical coupling. For example, CO_2_ and air temperature exhibited synchronized variability, whereas wind speed and air pressure showed a strong spatial anticorrelation, consistent with fundamental atmospheric dynamics (the pressure gradient force). These components were ranked by the proportion of variance they explained, and the top *p* components (*z*_1_ to *z_p_*) capturing more than 85% of the total variance was retained for regression analysis. We used the principal components (*z*_1_ to *z_p_*) as independent variables in the multiple regression and the normalized WUE_eco_ (y*) as the dependent variable

$$y^{*}=\beta_{0}^{*}+\beta_{1}^{*}z_{1}+\ldots +\beta_{p}^{*}z_{p}$$ (3)

By reversing the derivation process of the principal component analysis and *z*-score normalization (*54*), we derived the coefficients (β_1_ to β_7_) in the multiple regression equations for the original WUE_eco_ and climatic variables

$$y=\beta_{0}+\beta_{1}x_{1}+\ldots +\beta_{7}x_{7}$$ (4)

where the coefficient associated with wind speed represents the sensitivity of WUE_eco_ to wind (∂WUE_eco_/∂Wind).

The PCR approach reduces collinearity, improves model stability, and ensures that the dominant variability in the data is preserved. To test robustness, we repeated the analysis 1000 times for each grassland pixel using Monte Carlo resampling. The results showed a spatial mean relative uncertainty (SD/absolute mean value) of 12.55%, and an average coefficient of determination (*R*^2^) was 86.04% (fig. S15), underscoring that the sensitivity estimates are both precise and reliable.

We further calculated long-term trends in annual wind speed and WUE_eco_. The wind-induced WUE_eco_ increase was obtained by multiplying ∂WUE_eco_/∂Wind by the wind speed trend, and dividing this value by the overall WUE_eco_ increase gave the fractional contribution of wind speed. The same procedure was applied to the other six climatic variables.

Last, analysis of reanalysis data revealed that ∂WUE_eco_/∂Wind becomes more negative under drier SM conditions (Fig. 3D). To capture this dependency, we extended the PCR framework with an interaction term expressed as Wind/SM, providing a modified sensitivity metric [∂WUE_eco_/∂(Wind/SM)]. We chose the ratio form (Wind/SM) instead of the product (Wind × SM) because it better represents the amplified negative impact of wind speed on WUE_eco_ when SM declines.

### Wind manipulation simulation experiments using CLM5

To further test the robustness of the PCR-derived sensitivities and explore the mechanisms by which wind speed influences WUE_eco_, we carried out targeted wind manipulation experiments using CLM5, the land component of CESM2 (*55*). We selected the CLM5 model because it includes explicit representations of biophysical and biogeochemical processes. Specifically, wind speed directly modulates the boundary-layer conductance to water vapor (*g*_bw_), which governs the vapor pressure gradient and subsequent water exchange between the leaf surface and the atmosphere (*55*). This flux alteration triggers the model’s updated plant hydraulics module, which dynamically simulates leaf water potential and its feedback on stomatal behavior (*56*). The model-based perturbation experiments, following the experimental design established in our previous work (*14*), are essential for isolating the effects of wind speed on WUE_eco_ and for providing mechanistic support for our statistical findings.

We run all simulations in offline mode. Before the experiments, the model underwent a 400-year accelerated spin-up with enhanced soil organic matter decomposition, followed by an additional 800 years under standard conditions. This ensured that carbon, water, and energy fluxes reached equilibrium, with global ecosystem carbon drift (<0.02 Pg C year^−1^), well within the threshold recommended for C4MIP benchmarks (*57*).

Experimental simulations covered the period of 1983–2014 and included three scenarios. In the reference experiment, the model was driven by the default Climatic Research Unit–National Centers for Environmental Prediction (CRUNCEP) version 7 meteorological forcing (https://gdex.ucar.edu/datasets/d314003/), except that wind fields were replaced by CRU JRA version 2.2 data. This substitution was necessary because CRUNCEP showed an increasing wind trend inconsistent with observations, while CRU-JRA captured the observed decline. All forcing data had a 6-hourly temporal resolution and 0.5° by 0.5° spatial resolution, with simulation conducted at 0.9° by 1.25° resolution. Atmospheric CO_2_ concentration was fixed at 367 parts per million (default value in the model) in all simulations to isolate wind effects.

In the perturbation experiment, we uniformly adjusted wind speed by ±1 and ±8% across the entire simulation period, covering the observed range of terrestrial wind speed decline (−1.48 to −1.95% per decade) and reported declines up to 8% over recent decades (*11*). Intermediate perturbations were not included, as responses of WUE_eco_ to wind changes within this range were found to be linear (Fig. 2D).

In the detrended experiment, we removed wind speed trends using singular spectrum analysis (MATLAB’s “trenddecomp” function), applied at a 5-day resolution, instead of 6-hour or daily scale, to reduce the requirements of substantial computational resources.

From all simulations, we extracted WUE_eco_ (GPP/ET) and related biophysical variables including evaporation, transpiration, stomatal conductance, boundary-layer conductance to water vapor, relative humidity of canopy air, leaf temperature, and SM (0 to 29 cm) (*52*). Differences between reference and perturbation runs were calculated to quantify the impacts of wind changes, while detrended runs helped isolate the role of long-term wind change. To identify causal pathways linking wind speed to WUE_eco_, these variables were further integrated into an SEM framework. SEM analysis, implemented with the “lavaan” package in R, provided a satisfactory fit to the data, as evidenced by a comparative fit index value of 0.933, a standardized root mean square residual value of 0.066, a Tucker-Lewis index value of 0.817, and a root mean square error of approximation value of 0.105.
