# Supplementary material for: Wind stilling shapes grassland water use efficiency by enhancing soil moisture retention
Source: Sci Adv. 2026 May 13;12(20):eaee4995. doi: 10.1126/sciadv.aee4995 (PMC13170665; doi:10.1126/sciadv.aee4995)
Supplement: Supplementary file 1 — Figs. S1 to S15 Table S1 [file sciadv.aee4995_sm.pdf]

Science Advances
AAAS

Supplementary Materials for

Wind stilling shapes grassland water use efficiency by enhancing soil moisture retention

Haohao Wu *et al.*

Corresponding author: Congsheng Fu, csfu@niglas.ac.cn; Guishan Yang, gsyang@niglas.ac.cn

Sci. Adv. **12**, eaee4995 (2026)
DOI: 10.1126/sciadv.aee4995

This PDF file includes:

Figs. S1 to S15
Table S1

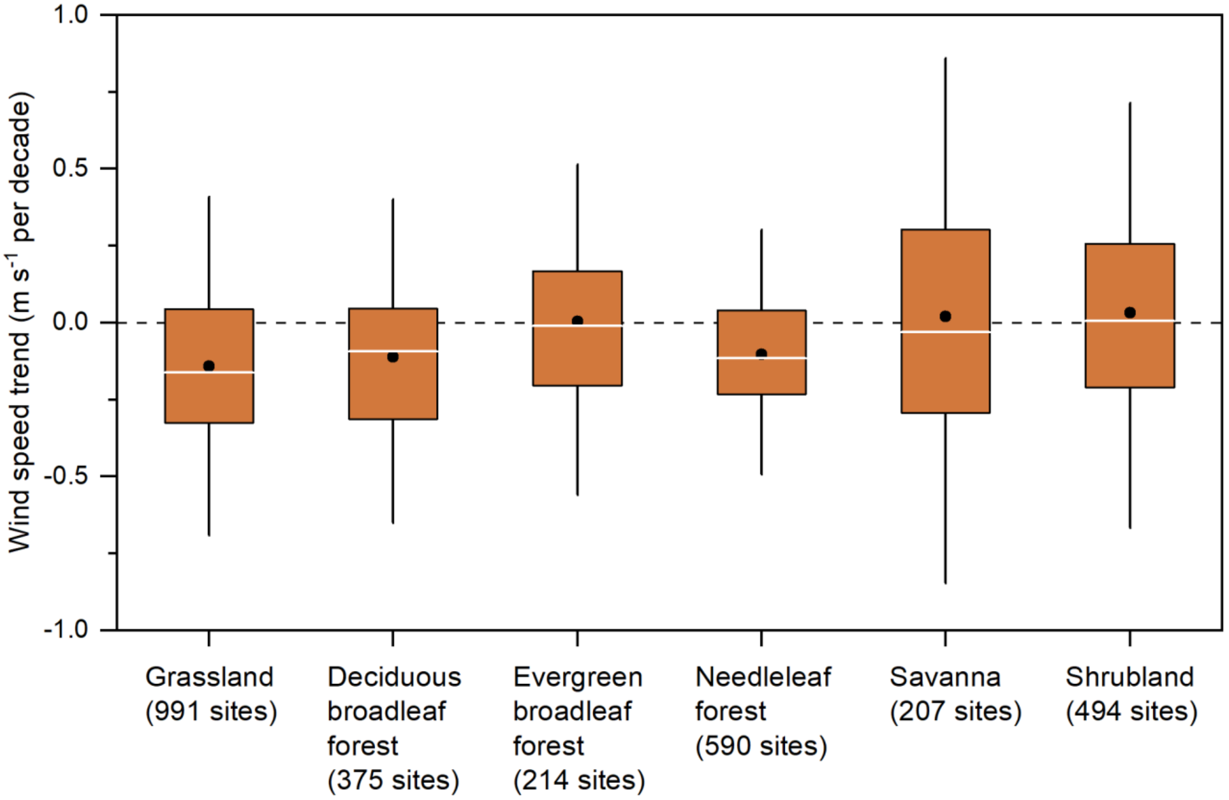

Fig. S1. Wind speed trends across different ecosystems during 1983–2010. Results are derived from observational data at 991 stations in the HadISD (v.3.4.1.2024f) global database.

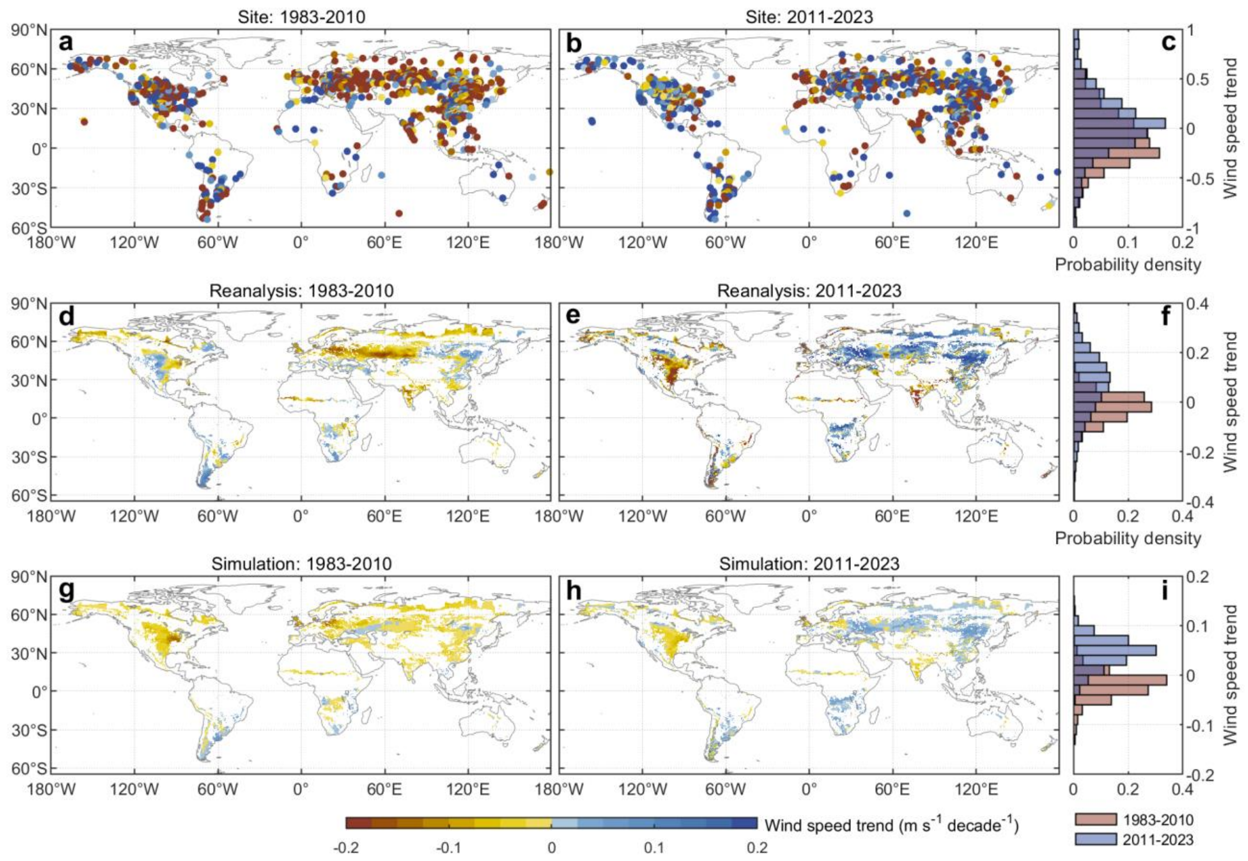

Fig. S2. Spatial distributions of wind speed trend during 1983–2010 and 2011–2023. Data are from 991 meteorological stations (**a–c**), reanalysis datasets (CRU, ERA5, and MERRA2) (**d–f**) and CMIP6 multi-model simulations (**h–i**). Panels **c**, **f** and **i** show probability densities of values in corresponding left panels.

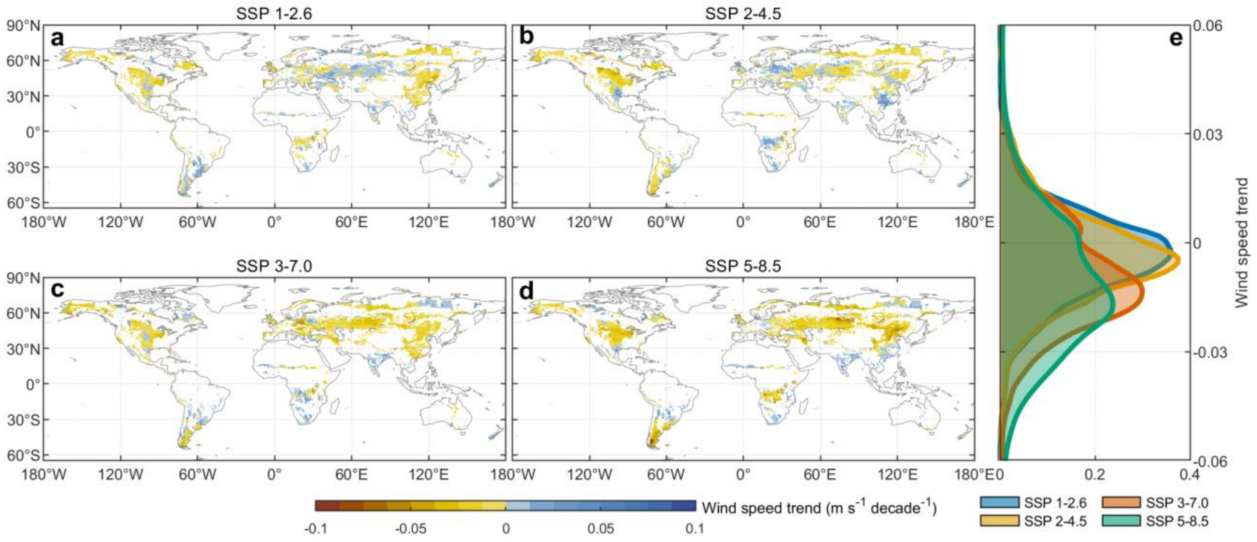

Fig. S3. Spatial distributions of wind speed trend during 2029–2100 under four future emission scenarios. Results are derived from simulations of six CMIP6 models. Panel **e** shows probability densities of wind speed trend in panels **a–d**.

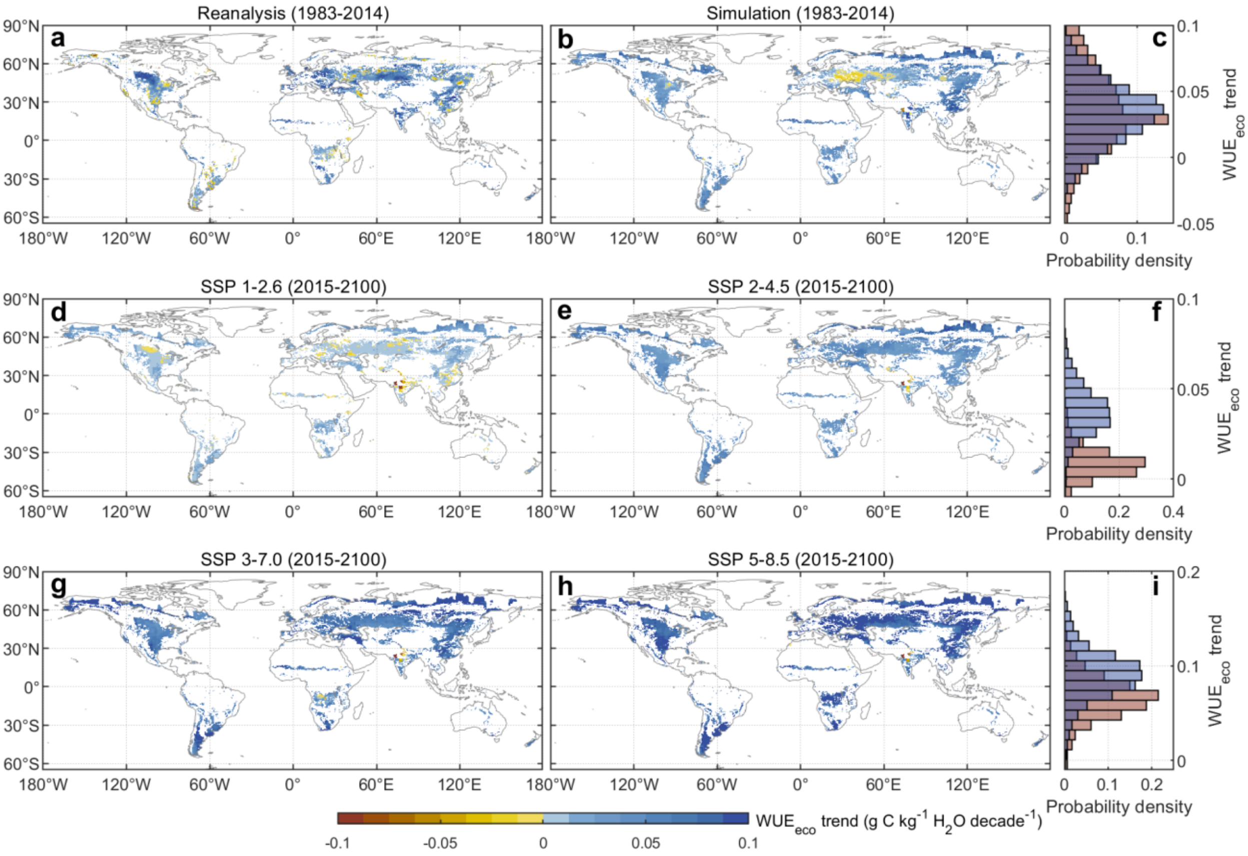

Fig. S4. Spatial distributions of historical and future trends of WUE_{eco}. Historical results in panel **a** are derived from reanalysis datasets, and historical and future results in panels **b–h** are derived from CMIP6 model simulations. Panels **c**, **f** and **i** show probability densities of values in corresponding left panels.

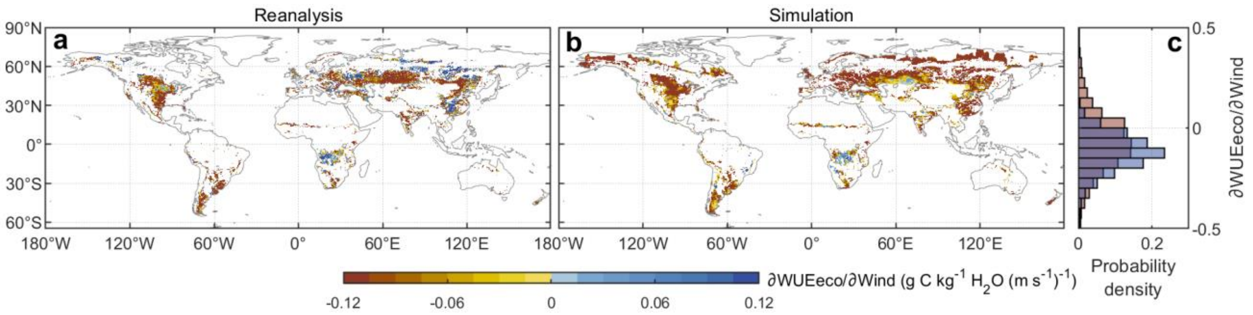

Fig. S5. Spatial distributions of ∂WUE_{eco}/∂Wind estimated from reanalysis datasets and CMIP6 simulations for the historical period (1983–2014). Panel **c** shows probability densities of values in panels **a** and **b**.

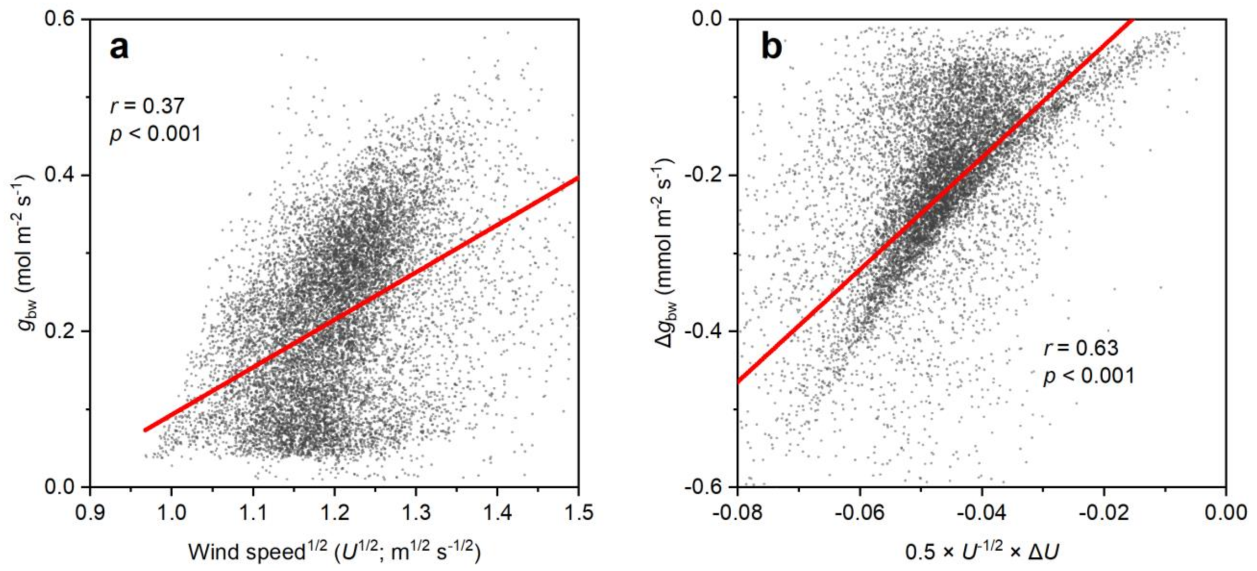

Fig. S6. Aerodynamic control of wind speed (*U*) on boundary-layer conductance to water vapor (g_{bw}). a, Relationship between g_{bw} and the square root of wind speed based on CLM5 control simulation; **b**, Relationship between Δg_{bw} and $0.5 \times U^{-1/2} \times \Delta U$. Δg_{bw} and ΔU were derived from CLM5 experiments (wind-speed-reduction run – control run); $0.5 \times U^{-1/2} \times \Delta U$ is the first-order theoretical predictor. Solid red lines denote linear regressions, with coefficients of determination (R^2) and significance levels (p) indicated.

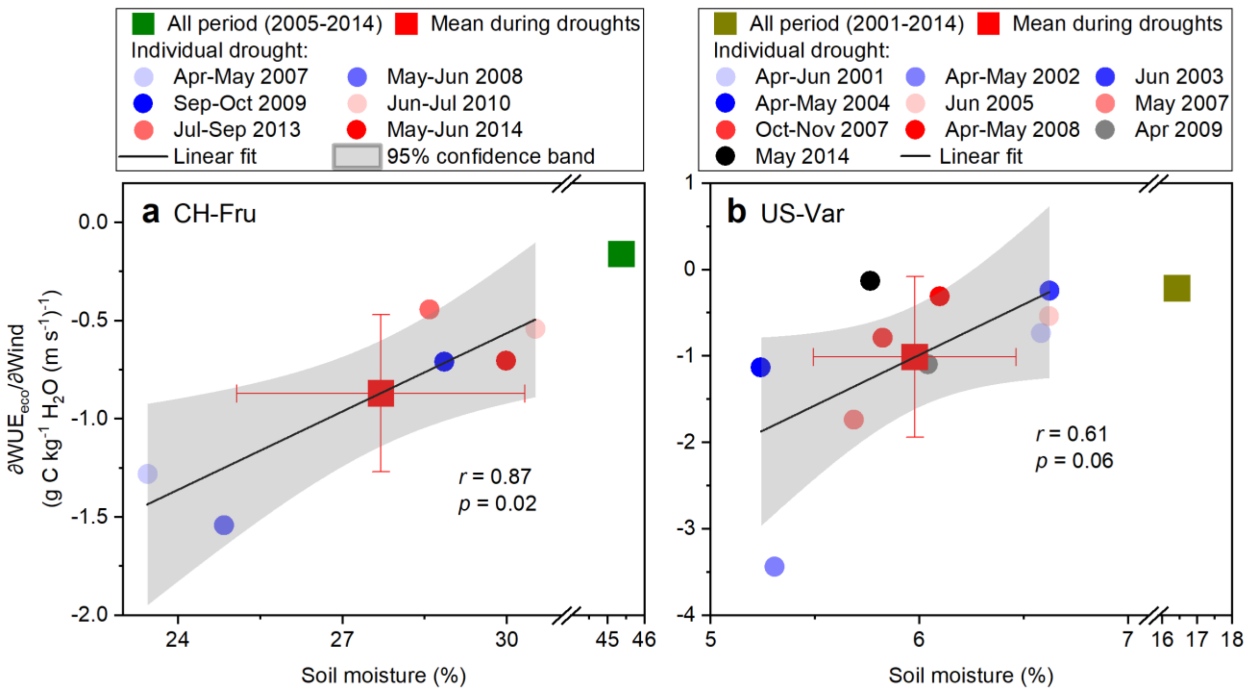

Fig. S7. Eddy covariance evidence for soil moisture regulation of ∂WUE$_{eco}$/∂Wind. Relationships between ∂WUE$_{eco}$/∂Wind and soil moisture at two representative FLUXNET grassland sites: **a**, CH-Fru and **b**, US-Var. Colored circles represent ∂WUE$_{eco}$/∂Wind plotted against mean soil moisture during individual drought periods. Red squares denote means across droughts, with error bars indicating ±1 one standard deviation. Green squares indicate estimates using the full observational period. Solid black lines show linear fits to drought period estimates, with gray shading indicating 95% confidence intervals. Pearson correlation coefficients (r) and corresponding p values are reported in each panel.

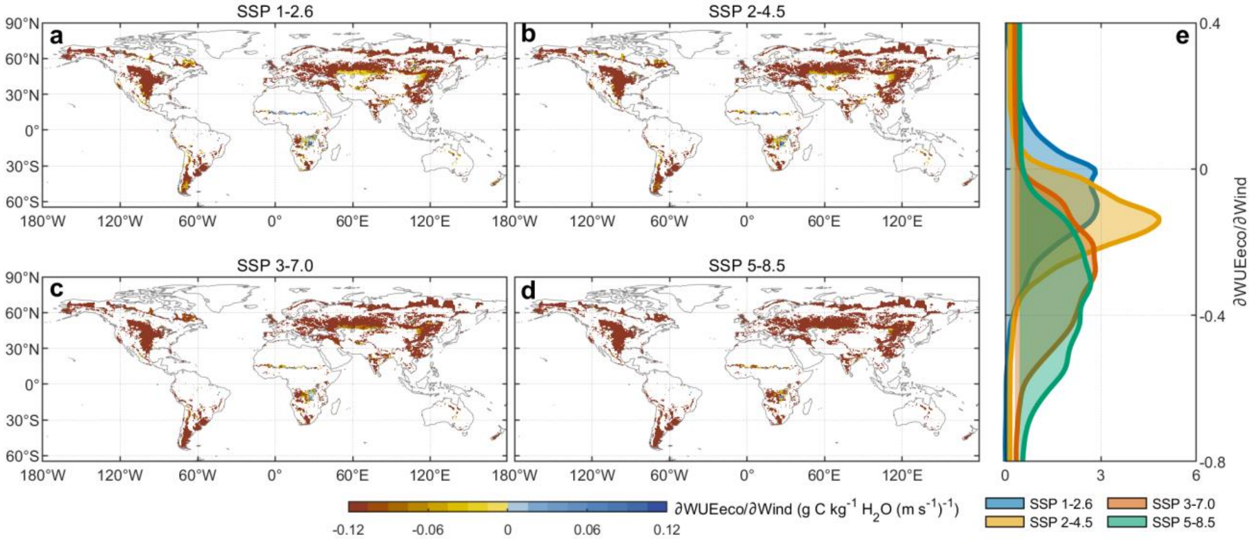

Fig. S8. Distributions of $\partial WUE_{eco}/\partial Wind$ during 2015–2100 estimated from CMIP6 simulations under four emission scenarios. Panel **e** shows probability densities of $\partial WUE_{eco}/\partial Wind$ in panels **a–d**.

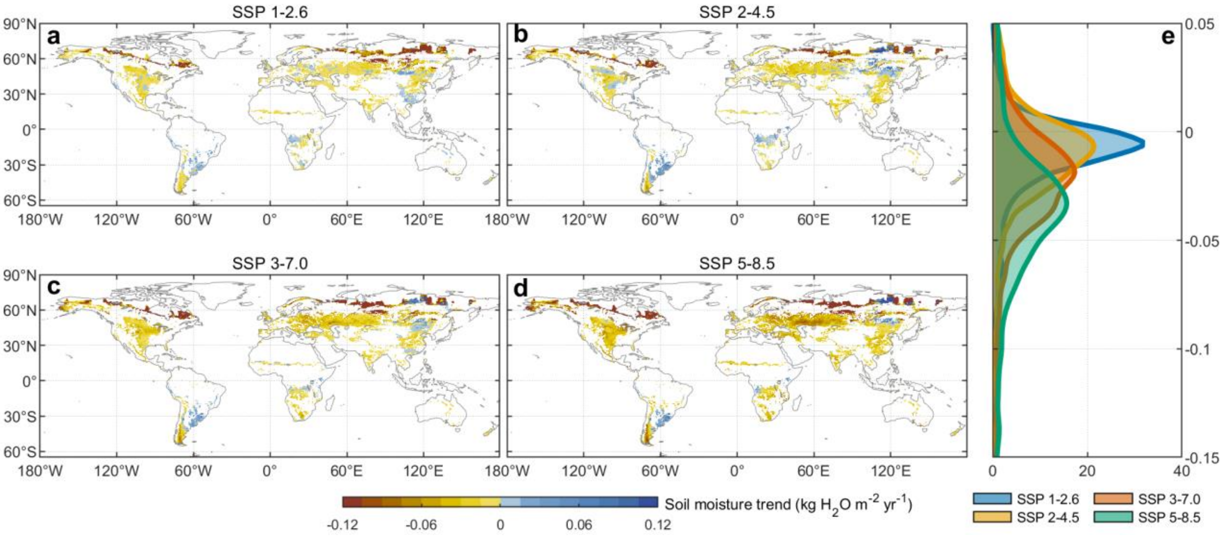

Fig. S9. Spatial distributions of soil moisture trend during 2015–2100 based on CMIP6 simulations under four future emission scenarios. Panel **e** shows probability densities of soil moisture trend in panels **a–d**.

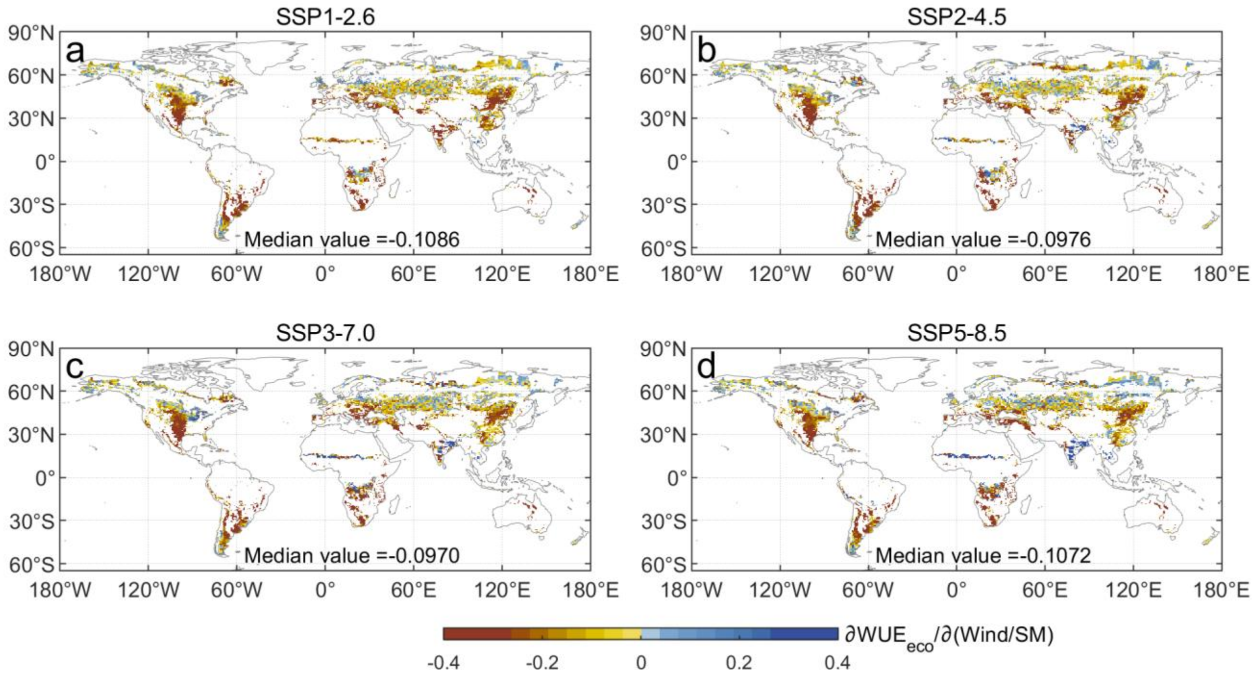

Fig. S10. Spatial distributions of the sensitivity of WUE_{eco} to interactions between wind speed and soil moisture ($\partial WUE_{eco}/\partial(Wind/SM)$) based on CMIP6 simulations under four future emission scenarios during 2015–2100 (a – d).

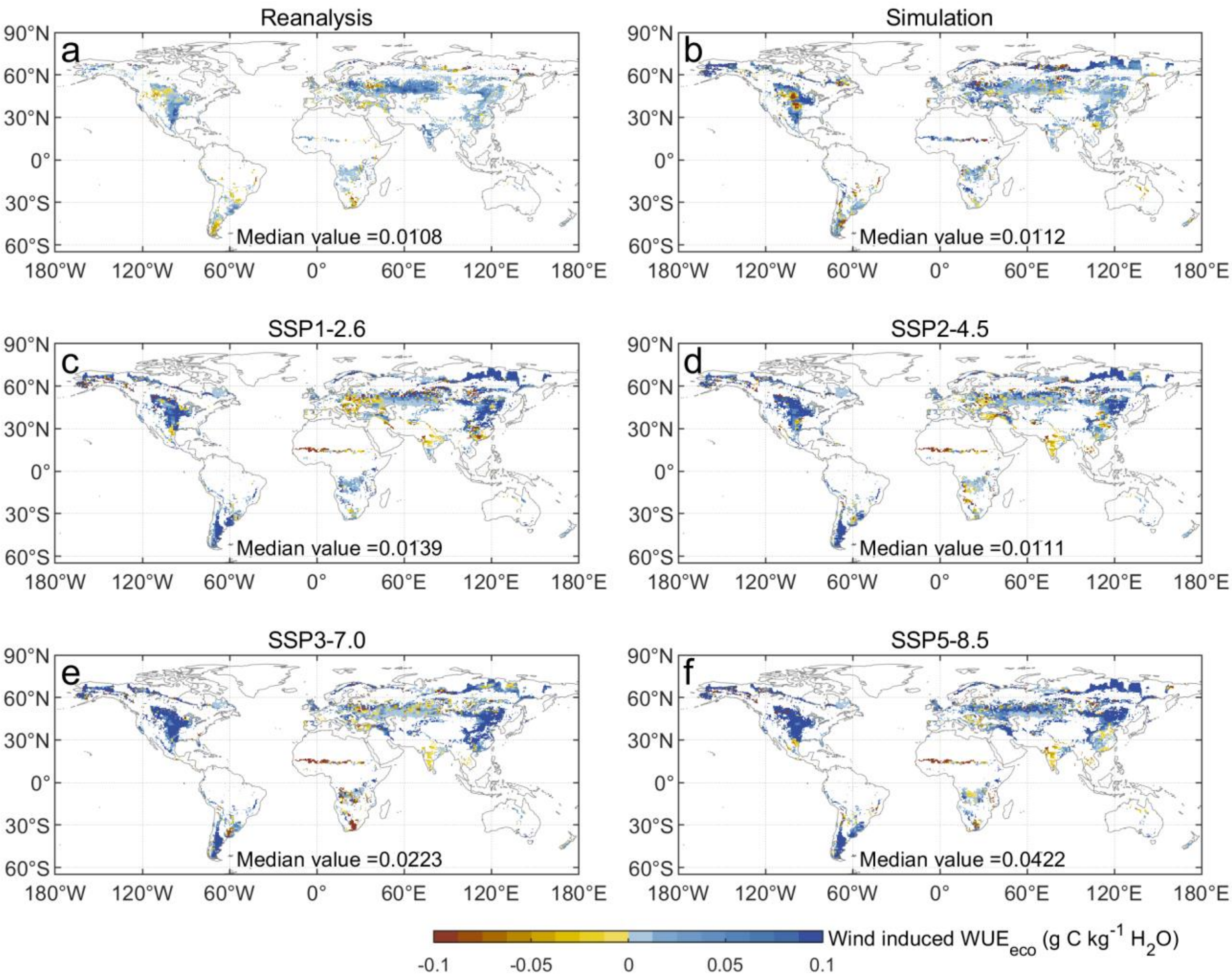

Fig. S11. Spatial distributions of wind induced WUE_{eco} increases estimated from reanalysis datasets (a) and CMIP6 simulations for the historical period (1983–2014; b) and for future projections (2015–2100; c–f) under the four emission scenarios.

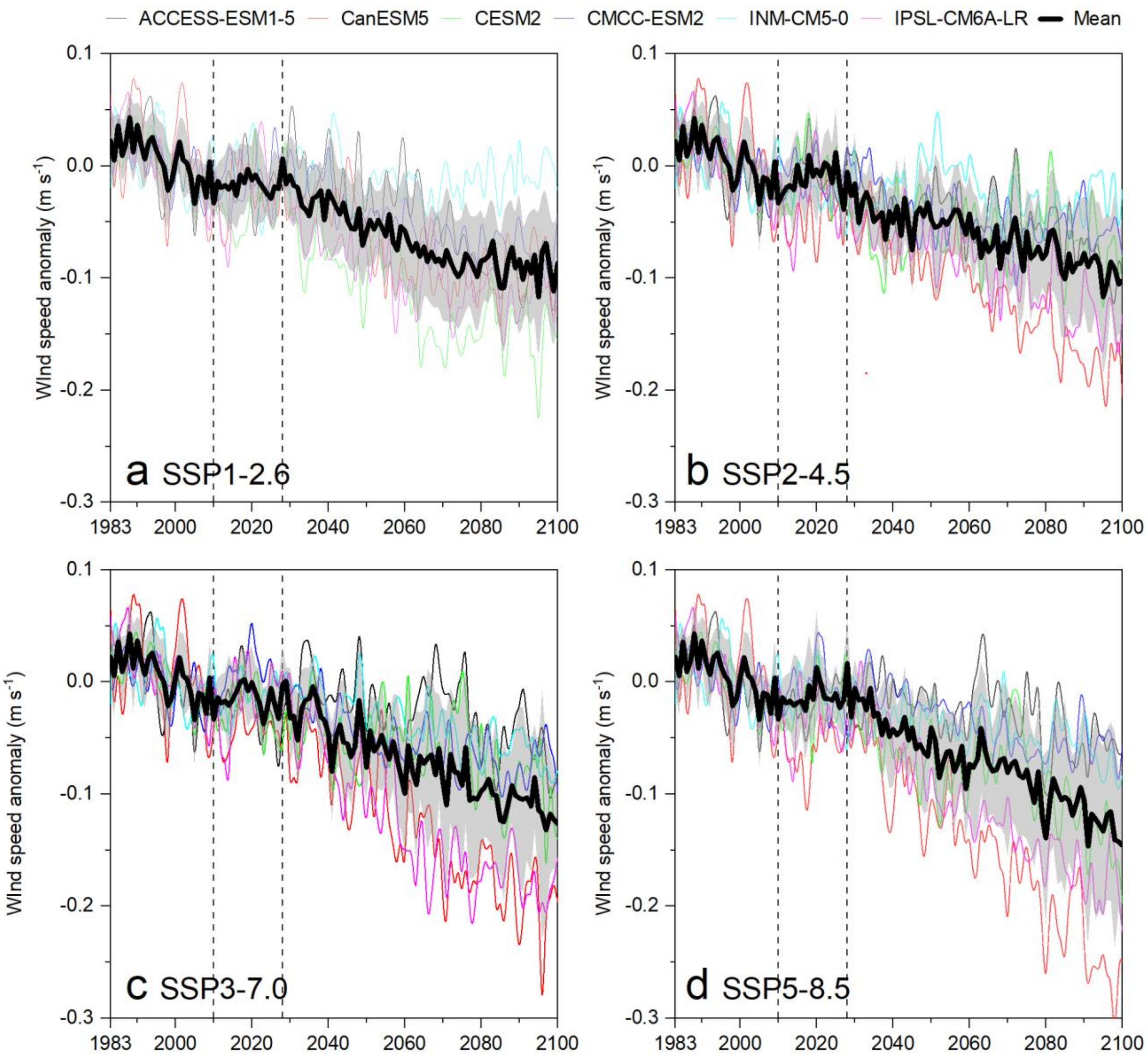

Fig. S12. Spatially averaged wind speed anomalies (1983–2100) from simulations of six CMIP6 models. Wind speed anomalies are shown for historical (1983–2014) and future (2015–2100) periods under four emission scenarios: SSP1-2.6 (**a**), SSP2-4.5 (**b**), SSP3-7.0 (**c**) and SSP5-8.5 (**d**). Note that **a–d** share the same historical simulations (1983–2014). The thick navy line in each panel represents the multi-model ensemble mean anomaly, and colored lines denote individual models.

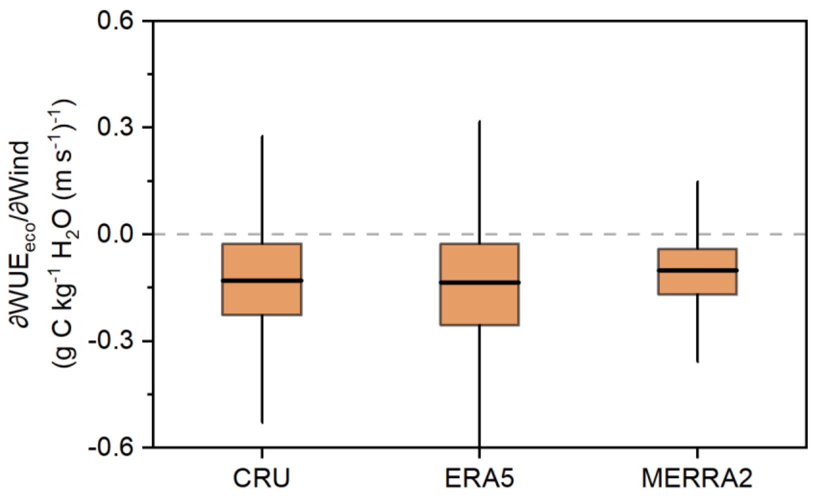

Fig. S13. Comparison of ∂WUE_{eco}/∂Wind derived from three independent reanalysis datasets. The box-and-whisker plots illustrate the global distribution of sensitivity values calculated using wind speed data from CRU, ERA5, and MERRA2 across all grassland pixels. The central horizontal line within each box represents the median, the box edges indicate the interquartile range (IQR, 25th to 75th percentiles), and the whiskers extend to 1.5 times the IQR.

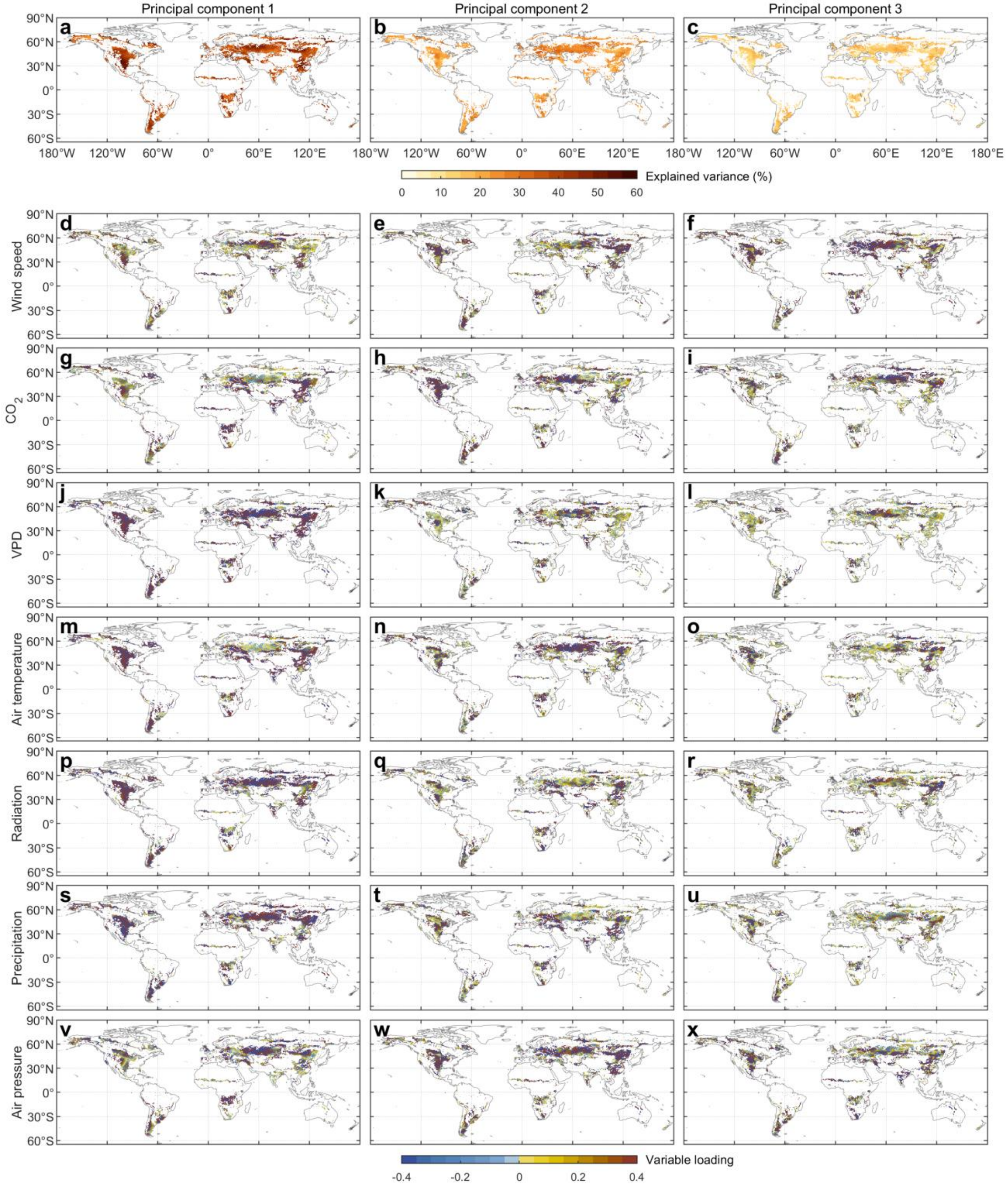

Fig. S14. Spatial patterns of PCA characteristics. The top row (**a**–**c**) displays the explained variance (%) of the first three principal components of the seven climatic variables, reflecting the

relative importance of each component in capturing climatic variability. The subsequent rows (**d**–**x**) illustrate the loading factors of individual climatic variables for each principal component. The PCA results were derived from ERA5 climatic datasets during 1983–2014.

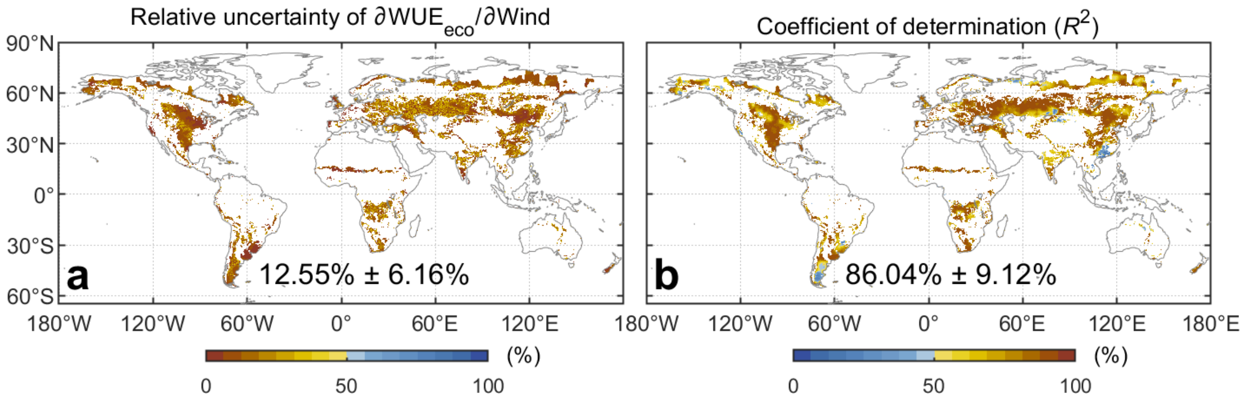

Fig. S15. The performance of the PCR approach in estimating ∂WUE$_{eco}$/∂Wind. The spatial distributions of the relative uncertainty (i.e., standard deviation ÷ absolute mean value) of ∂WUE$_{eco}$/∂Wind based on 1000 Monte Carlo simulations and the coefficient of determination of the PCR fitting used in the study are shown in panels (**a**) and (**b**), respectively.

Table S1. Information and estimated $\partial WUE_{eco}/\partial Wind$ at selected FLUXNET sites.

Sites	Latitude (°)	Longitude (°)	Observation years	$\partial WUE_{eco}/\partial Wind$ (g C m^{-2} yr^{-1} (m $s^{-1})^{-1}$)
AT-Neu	47.12	11.32	2002–2012	-1.32
AU-Stp	-17.15	133.35	2008–2014	0.01
CH-Cha	47.21	8.41	2005–2014	-0.13
CH-Fru	47.11	8.54	2005–2014	-0.16
CH-Oe1	47.29	7.73	2002–2008	0.32
Cz-BK2	49.49	18.54	2004–2012	-0.47
DE-Gri	50.95	13.51	2004–2014	-0.33
IT-MBo	46.01	11.05	2003–2013	-0.04
IT-Tor	45.84	7.58	2008–2014	0.03
US-IB2	41.84	-88.24	2004–2011	-0.05
US-SRG	31.79	-110.83	2008–2014	-0.15
US-Var	38.41	-120.95	2000–2014	-0.11
US-Wkg	31.74	-109.94	2004–2014	0.07
